# Breastfeeding practices and policies in WHO European Region Member States

**DOI:** 10.1017/S1368980015001767

**Published:** 2015-06-22

**Authors:** Ayse Tulay Bagci Bosi, Kamilla Gehrt Eriksen, Tanja Sobko, Trudy MA Wijnhoven, João Breda

**Affiliations:** 1 Department of Public Health, School of Medicine, Hacettepe University, Ankara, Turkey; 2 MRC Human Nutrition Research, Darwin College, Cambridge, Silver Street, Cambridge CB3 9EU, UK; 3 Department of Human Performance, Hong Kong University, Pokfulam, Hong Kong SAR, People’s Republic of China; 4 Division of Noncommunicable Diseases and Promoting Health through the Life-Course, WHO Regional Office for Europe, Copenhagen, Denmark

**Keywords:** Breastfeeding, Policy, Baby-friendly hospitals, WHO European Member States

## Abstract

**Objective:**

To provide an update on current practices and policy development status concerning breastfeeding in the WHO European Region.

**Design:**

National surveys and studies conducted by national health institutions were prioritized. Sub-national data were included where no national data or studies existed. Information on national breastfeeding policies was collected mainly from the WHO Seventh Meeting of Baby-Friendly Hospital Initiative Coordinators and European Union projects. Owing to the different data sources and methods, any comparisons between countries must be made with caution.

**Setting:**

WHO European Member States.

**Results:**

Data from fifty-three WHO European Member States were investigated; however, a large proportion had not reported any data. Rates of early initiation of breastfeeding, exclusive breastfeeding and continued breastfeeding to 1 year all varied considerably within the WHO European Region. Exclusive breastfeeding rates declined considerably after 4 months, and were low in infants under 6 months and at 6 months of age. The majority of the countries with existing data reported having a national infant and young child feeding policy and the establishment of a national committee on breastfeeding or infant and young child feeding. The majority of the countries with existing data reported having baby-friendly hospitals, although the proportion of baby-friendly hospitals to the total number of national hospitals with maternity units was low in most countries.

**Conclusions:**

Breastfeeding practices within the WHO European Region, especially exclusive breastfeeding rates, are far from complying with the WHO recommendations. There are marked differences between countries in breastfeeding practices, infant and young child feeding policy adoption and proportion of baby-friendly hospitals.

Breastfeeding is widely recognized as the best option for infant feeding and is considered a critical element for public health, not only a matter of lifestyle choice^(^ ^1^ ^,^ ^2^ ^)^. The short- and long-term health benefits of breastfeeding for children and mothers have been well documented^(^ ^3^ ^–^ ^5^ ^)^. For instance, breastfeeding has psychological, economic and environmental benefits^(^ ^6^ ^)^. Optimal breastfeeding practices are associated with a lower risk of childhood gastrointestinal infections, otitis media, asthma, respiratory disease and sudden infant death syndrome^(^ ^7^ ^,^ ^8^ ^)^. Moreover, research evidence shows the protective effect of breastfeeding on the incidence of non-communicable diseases, notably obesity, CVD and diabetes mellitus^(^ ^7^ ^,^ ^9^ ^–^ ^12^ ^)^. Mechanisms explaining the association between breastfeeding and obesity have been summarized in a recent review^(^ ^13^ ^)^.

The evidence suggests that early nutrition factors such as breastfeeding may present a ‘window of opportunity’ for obesity policy prevention^(^ ^14^ ^)^, to respond to the problem of childhood obesity in Europe^(^ ^15^ ^)^. For example, an estimated 19–49 % of European school-aged boys and 18–43 % of girls are overweight. The prevalence of obesity ranges from 6 to 27 % among boys and from 5 to 17 % among girls^(^ ^16^ ^)^. In addition, studies suggest that overweight or obese children are more likely to be overweight or obese adults^(^ ^17^ ^)^, which may have adverse morbidity and mortality impact later in life^(^ ^18^ ^)^. Furthermore, obesity is already responsible for 2–8 % of health costs and 10–13 % of deaths in Europe^(^ ^19^ ^)^. Given the documented health and economic consequences, and limited success in obesity treatment^(^ ^20^ ^)^, protection and promotion of breastfeeding has been identified as an important public health priority to support appropriate nutrition in early life as well as to contribute to the prevention of childhood obesity and other non-communicable diseases later in life^(^ ^21^ ^–^ ^24^ ^)^.

The WHO recommends exclusive breastfeeding (EBF) for the first 6 months of life and introduction of complementary food after 6 months along with continued breastfeeding up until 2 years or beyond^(^ ^25^ ^)^. This global recommendation of EBF is highly supported by international communities, organizations and scientists, and the promotion, protection and support of breastfeeding has become a public health priority^(^ ^6^ ^,^ ^26^ ^)^. Member States of WHO adopted in 2002 the Global Strategy for Infant and Young Child Feeding (hereafter, the ‘Global Strategy’), which advocates for comprehensive national policies that promote, protect and support infant and young child feeding (IYCF) practices. The Global Strategy component includes the International Code of Marketing of Breastmilk Substitutes^(^ ^27^ ^)^. Moreover, the Innocenti Declaration^(^ ^28^ ^)^, the Baby Friendly Hospitals Initiative (BFHI)^(^ ^29^ ^)^, the WHO Maternal, Infant and Young Child Nutrition Implementation Plan^(^ ^30^ ^)^ and the World Health Assembly Global Targets for Nutrition 2025^(^ ^31^ ^)^ collectively play an important role in increasing breastfeeding prevalence and monitoring progress.

Despite these political commitments, breastfeeding prevalence – especially EBF – remains low in the WHO European Region. In 2006–2012, only an estimated 25 % of infants were exclusively breastfed for the first 6 months in the WHO European Region as compared with 43 % in the WHO South-East Asia Region^(^ ^32^ ^)^. Studies have also found wide disparities in breastfeeding prevalence, weaning practices and adoption of the Global Strategy among WHO European Region Member States^(^ ^33^ ^–^ ^35^ ^)^. One of the most recent estimations of breastfeeding practices and policy development status in twenty-nine European countries was produced back in 2007^(^ ^33^ ^)^. However since then, a broad range of policy initiatives and activities were introduced in several of the WHO European Region Member States that may have influenced breastfeeding practices as well as the status of the national policies to increase breastfeeding rates.

Although the beneficial effect of breastfeeding in reducing the risk of infant morbidity has been widely accepted^(^ ^7^ ^,^ ^8^ ^)^, collection of data on breastfeeding practices continues to be rather limited in the WHO European Region. Additionally, information on IYCF practices is scarce in some of the international databases and other sources, although more data on breastfeeding practices in European countries have become available recently^(^ ^36^ ^)^. Up-to-date information on breastfeeding practices is essential to track the progress among countries, which may better equip policy-making bodies for their evaluation of policy development and implementation of policy actions. As such, the present work aims to provide an update on current practices and policy status concerning breastfeeding in the WHO European Region.

## 

### Definitions used in the present paper

#### Breastfeeding practices

WHO recommends use of three core indicators for assessing breastfeeding practices, i.e. early initiation of breastfeeding, exclusive breastfeeding and continued breastfeeding at 1 year. The early initiation of breastfeeding is defined as the proportion of children born in the last 24 months who were put to the breast within 1 h of birth. Similarly, exclusive breastfeeding under 6 months is the proportion of infants 0–5·9 months of age who were fed exclusively with breast milk. Continued breastfeeding at 1 year is the proportion of children 12–15·9 months of age who were fed breast milk^(^ ^37^ ^)^.

#### Breastfeeding policy

A formal document setting out the government’s position on the recommended breastfeeding practices and the principle of action to achieve national goals, and generally is developed over a long time period while it is cleared with relevant bodies^(^ ^38^ ^)^.

#### Baby-friendly hospital

A hospital or maternity facility can be designated as a baby-friendly hospital (BFH) when it does not accept free or low-cost breast milk substitutes, feeding bottles or teats, and has implemented ten specific steps to support successful breastfeeding^(^ ^29^ ^)^.

## Methods

Breastfeeding data were collected according to the three WHO recommended practices of breastfeeding. Additionally, EBF under 4 months was included, as a majority of WHO European Region Member States reported data on EBF at 0–3·9 months of age. Data on EBF at 6 months were furthermore included in the present paper, as many countries reported such data instead of EBF under 6 months.

Emphasis has been placed on collecting data from national surveys or studies done by national health institutions. Where national representative surveys were lacking, intervention studies, birth cohort studies and data from the WHO global databank on IYCF and the Organisation for Economic Co-operation and Development (OECD) family database were included. For this reason, data should be interpreted with caution and the comparability is limited. A combination of search methods was employed up to December 2013 that included an Internet-based search in official websites of national ministries of health, ministries of women’s and children’s affairs, and different national and international breastfeeding promotion agencies. To assess data available in original languages, Google Translate was used. Furthermore, the state-of-the-art literature related to breastfeeding in the fifty-three Member States of the WHO European Region was searched and assessed via the PubMed database and Google Scholar by using the following keywords: ‘breastfeeding’ AND/OR ‘name of the country’; ‘infant and young child feeding practices’; ‘breastfeeding prevalence’ and ‘breastfeeding statistics’. In addition, the WHO global databank on IYCF, the UNICEF database monitoring the situation of children and mothers, the OECD family database and the International Baby Food Action Network (IBFAN) statistics were used to retrieve up-to-date information. The national surveys and studies used in the present paper were published in 2003–2013, with data collected between 1998 and 2013. One exception on this was a study carried out in the Republic of Moldova in 2012, of which its report was published in 2014. A full reference list of the data used is provided in the Appendix.

Information on national breastfeeding policies was also obtained from the WHO Country Reports, which were based on a Member State Survey between 2000 and 2011 and addressed at the Seventh Meeting of BFHI Coordinators of Industrialized Countries in 2012^(^ ^39^ ^)^. The meeting report presents the information on current status of breastfeeding related policies and BFH as reported in the questionnaire by Member States. The additional and missing information was sourced from Promotion of Breastfeeding in Europe: Pilot Testing the Blueprint for Action Project (2008)^(^ ^26^ ^)^ and Promotion of Breastfeeding in Countries Wishing to Join the European Union (2004)^(^ ^40^ ^)^. Similarly up-to-date data on BFH were adopted from the WHO European Database for Nutrition, Obesity and Physical Activity (NOPA)^(^ ^41^ ^)^.

## Results

A summary of collected data on breastfeeding practices is presented in Table 1. In total, data from forty-five WHO European Member States were identified. Among these, thirty-nine were national data and six were other studies (including sub-national studies and peer-reviewed articles). Of the national data, 15 % were Demographic and Health Surveys (DHS), 21 % were Multiple Indicator Cluster Surveys (MICS) and 64 % were surveys conducted by national ministries of health.

**Table 1:** Overview of collected data on breastfeeding practices in the WHO European Region

Type of study	Country (*n*)	%
National data	39	74
Demographic and Health Survey (DHS)	6	
Multiple Indicator Cluster Survey (MICS)	8	
National surveys other than DHS and MICS	25	
Other studies*	6	11
No data	8	15
Total	53	100

* Other studies include sub-national studies and peer-reviewed articles.

An overview of breastfeeding practices in the WHO European Region is given in Table 2. Wide variation was noted among the countries in reporting data on recommended practices, as seen in Table 3. Twenty-one out of fifty-three countries reported data on early initiation of breastfeeding, all within 1 h after birth except Austria that reported on breastfeeding within 1–2 h after birth. Thirteen countries reported data on EBF under 4 months and data on EBF under 6 months were available for twenty-four countries. Data on EBF at 6 months were available for twenty-one countries, whereas twenty-five countries had data on continued breastfeeding rate at 1 year. It was investigated if any relationship existed between high data availability and high prevalence of EBF patterns. It was found that countries with data for at least four out of the five indicators presented in Table 3 had a median rate of EBF under 6 months of 30 % (ten countries) and countries with data available for three or fewer had a median rate of EBF under 6 months of 21 % (fourteen countries).

**Table 2:** Overview of breastfeeding practices in the WHO European Region* (data from 1998 to 2013)

	Breastfeeding within 1 h after birth (%)	EBF under 4 months (0–3·9 months; %)	EBF under 6 months (0–5·9 months; %)	EBF at 6 months (%)	Continued breastfeeding at 1 year (%)	Year of data collection
Albania	42·9	50·2	38·6	–	60·6	2008–2009
Armenia	35·7	47·9	35·0	–	44·2	2010
Austria	78·1	33·0	32·6	10·0	16·0	2006
Azerbaijan	31·9	16·0	11·8	–	26·4	2006
Belarus	53·0	–	19·0	–	27·9	2012
Belgium	–	–	–	11·8	–	2012
Bosnia and Herzegovina	42·3	23·6	18·5	–	12·4	2006/2011–2012
Bulgaria	4·6	5·7	2·0	–	–	2010
Croatia	–	–	52·4	–	–	2011
Cyprus	–	–	–	12·4	–	2004
Czech Republic	–	–	–	17·8	–	2011
Denmark	–	–	–	17·2	–	2012
Finland	–	–	–	1·0	–	2011
Georgia	66·3	–	54·8	–	36·5	2009
Germany	–	–	22·4	–	–	2003–2006
Greece	–	–	–	0·7	6·4	2009
Hungary	–	–	–	43·9	–	2007
Iceland	–	–	–	13·0	16·0	2011
Ireland	33·5	–	–	–	–	2008
Israel	–	–	–	11·2	11·8	1998–1999
Italy	–	–	–	5·0	12·0	1999
Kazakhstan	67·8	–	31·8	–	50·8	2010–2011
Kyrgyzstan	83·8	66·2	56·1	–	68·3	2012
Latvia	–	–	–	16·4	22·4	2011
Luxembourg	66·5	–	–	6·0	11·8	2008
Malta	–	–	–	35·9	–	2004–2005
Montenegro	25·2	25·8	19·3	–	24·6	2005
Netherlands	–	–	–	18·0	–	2010
Norway	–	–	–	7·0	–	2003
Poland	–	–	3·7	–	40·0	2013
Portugal	–	54·7	–	34·0	–	2003
Republic of Moldova	61·0	–	36·0	–	48·0	2012
Romania	12·0	–	15·8	–	–	2004
Serbia	7·6	23·4	13·7	–	18·4	2005–2006/2010
Slovakia	–	–	–	49·3	–	2010
Spain	–	–	–	28·5	–	2011–2012
Sweden	–	–	–	14·0	–	2011
Switzerland	–	–	14·0	–	–	2003
Tajikistan	49·6	42·1	34·3	–	1·3	2012
The former Yugoslav Republic of Macedonia	21·0	–	23·0	–	33·8	2011
Turkey	39·0	–	41·6	–	66·7	2008
Turkmenistan	–	15·0	11·0	–	72·0	2009
Ukraine	65·7	–	19·7	–	37·9	2012
UK	–	–	–	1·0	–	2010
Uzbekistan	67·1	36·9	26·4	–	78·3	2006

EBF, exclusive breastfeeding; –, no data.

* No data for Andorra, Estonia, France, Lithuania, Monaco, Russian Federation, San Marino and Slovenia.

**Table 3:** Summary of reporting countries and proportion of breastfeeding practices in the WHO European Region (data from 1998 to 2013)

	Reporting countries			
Indicator	*n*	%	Minimum–maximum (%)	Median (%)
Breastfeeding within 1 h after birth*	21	40	5–84	43
EBF under 4 months (0–3·9 months)	13	25	6–66	33
EBF under 6 months (0–5·9 months)	24	45	2–56	23
EBF at 6 months†	21	40	1–49	13
Continued breastfeeding at 1 year (12–15·9 months)‡	25	47	1–78	28

EBF, exclusive breastfeeding.

* Data for Austria are included and reported on breastfeeding within 1–2 h after birth.

† Austria was the only country that reported data on both EBF under 6 months and EBF at 6 months.

‡ Data reported at 12 months are included in this category.

### Early initiation of breastfeeding within 1 h after birth

The rates of early initiation of breastfeeding within 1 h after birth varied widely across the WHO European Region Member States (Table 2). As minimum and maximum, 5–84 % (median 43 %) of infants were breastfed within 1 h after birth, based on data from twenty-one out of fifty-three countries (Table 3). Nine of the twenty-one countries had an initiation rate above 50 %. The lowest prevalence was seen in Bulgaria (5 %) and Serbia (8 %), and the highest prevalence in Kyrgyzstan, where 84 % of infants had initiated early breastfeeding 1 h after birth.

### Exclusive breastfeeding under 4 months

The rates of EBF of infants under 4 months varied widely across the WHO European Region Member States (Table 2). As minimum and maximum, 6–66 % (median 33 %) of infants under 4 months of age were exclusively breastfed, based on data from thirteen out of fifty-three countries (Table 3). Only three out of these thirteen countries had EBF rates of more than 50 % for infants <4 months old. The highest rate was reported in Kyrgyzstan (66 %) and the lowest rate was found in Bulgaria (6 %).

### Exclusive breastfeeding under 6 months and at 6 months

The proportion of infants being exclusively breastfed decreased with age. The minimum and maximum prevalence of EBF under 6 months was 2–56 % (median 23 %) based on data from twenty-four out of fifty-three countries (Table 3). Only thirteen out of these twenty-four countries reported prevalence rates above 20 % (Table 2). Higher rates appeared in Kyrgyzstan (56 %), Georgia (55 %) and Croatia (52 %), with lower rates reported from Poland (4 %) and Bulgaria (2 %). Regarding breastfeeding at 6 months, twenty-one countries out of fifty-three reported these data, with a minimum and maximum of 1–49 % (median 13 %) of infants being exclusively breastfed at 6 months (Table 3). The highest rate was seen in Slovakia (49 %) and Hungary (44 %) and the lowest in Greece (1 %), Finland (1 %) and the UK (1 %).

### Continued breastfeeding at 1 year

Table 2 shows the proportion of children who were still breastfed at 1 year in the WHO European Region. As minimum and maximum, 1–78 % (median 28 %) of infants were breastfed at 1 year, based on data from twenty-five out of fifty-three countries (Table 3). Higher rates of continued breastfeeding at 1 year were found in Uzbekistan (78 %) and Turkmenistan (72 %), whereas Greece (6 %) and Tajikistan (1 %) reported the lowest rates among the twenty-five countries.

### Breastfeeding policy status

The data on existing policy status were available for thirty-four countries out of fifty-three WHO European Member States. The year of data collection is presented in Table 4. Data extracted from the NOPA database included Albania (2003), Armenia (1999), Bosnia and Herzegovina (2002), Kazakhstan (2010) and Turkey (2004). Table 4 highlights that twenty-three countries out of thirty-four reported having either a national policy on IYCF or a nutrition policy or other policies that include IYCF (68 %). Data were missing for nineteen countries. Concerning the national committee on breastfeeding or IYCF, sixteen out of twenty-four (67 %) reported an established committee. No data were available for twenty-nine countries.

**Table 4:** Overview of existing breastfeeding or IYCF policies and BFH in WHO European Member States (data from 1999 to 2012)

	Policies		BFH*		
Country (year of data collection)	National breastfeeding, IYCF or nutrition policy† (yes/no)	National breastfeeding or IYCF committee‡ (yes/no)	Total hospitals with maternity units (*n*)	Ever designated (*n*)	%
Albania (2003)	Yes	N/D	N/D	N/D	
Armenia (1999)	Yes	N/D	N/D	N/D	
Austria (2012§)	Yes	Yes	79	17	21·5
Belgium (2012§)	Yes	Yes	107	22	20·6
Bosnia and Herzegovina (2002)	Yes	N/D	N/D	N/D	
Bulgaria (2004)	Yes	Yes	N/D	5	N/D
Croatia (2012§)	Yes	Yes	32	21	65·6
Czech Republic (2007)	No	N/D	105	58	55·2
Denmark (2012§)	N/D	Yes	24	12	50·0
Estonia (2004)	Yes	No	17	1	5·8
Finland (2012§)	Yes	Yes	31	5	16·1
France (2012§)	No	No	533	17	3·2
Germany (2012§)	Yes	Yes	925	70	7·6
Greece (2007)	Yes	N/D	34	0	
Hungary (2004)	No	Yes	100	9	9·0
Iceland (2007)	No	N/D	15	0	
Ireland (2012§)	No	No	20	9	45·0
Italy (2012§)	Yes	Yes	560	24	4·3
Kazakhstan (2010)	Yes	N/D	N/D	N/D	
Latvia (2007)	Yes	N/D	30	14	46·7
Lithuania (2012§)	Yes	Yes	35	8	22·9
Luxembourg (2012§)	Yes	Yes	4	3	75·0
Malta (2012§)	No	No	4	0	
Netherlands (2010)	N/D	No	92	63	68·5
Norway (2012§)	Yes	Yes	47	43	91·5
Poland (2012§)	N/D	N/D	450	89	19·8
Portugal (2007)	No	N/D	60	1	1·7
Romania (2007)	Yes	N/D	204	30	14·7
Russian Federation (2012§)	Yes	Yes	3000	287	9·6
Slovakia (2004)	Yes	N/D	72	21	29·2
Slovenia (2012§)	Yes	Yes	14	13	92·9
Spain (2012§)	No	No	298	17	5·7
Sweden (2012§)	No	Yes	52	33	63·5
Switzerland (2012§)	No	No	300	75	25·0
Turkey (2004)	Yes	N/D	N/D	N/D	
Ukraine (2012§)	Yes	Yes	667	396	59·4
UK (2012§)	No	No	459	N/D	

IYCF, infant and young child feeding; BFH, baby-friendly hospital; N/D, no data.

* No data from Albania, Andorra, Armenia, Azerbaijan, Belarus, Bosnia and Herzegovina, Cyprus, Georgia, Israel, Kazakhstan, Kyrgyzstan, Monaco, Montenegro, Republic of Moldova, San Marino, Serbia, Tajikistan, the former Yugoslav Republic of Macedonia, Turkey, Turkmenistan, UK and Uzbekistan.

† No data from Andorra, Azerbaijan, Belarus, Cyprus, Georgia, Israel, Kyrgyzstan, Monaco, Montenegro, Netherlands, Poland, Republic of Moldova, San Marino, Serbia, Tajikistan, the former Yugoslav Republic of Macedonia, Turkmenistan and Uzbekistan.

‡ No data from Albania, Andorra, Armenia, Azerbaijan, Belarus, Bosnia and Herzegovina, Cyprus, Georgia, Greece, Iceland, Israel, Kazakhstan, Kyrgyzstan, Monaco, Montenegro, Poland, Republic of Moldova, Romania, San Marino, Serbia, Slovakia, Tajikistan, the former Yugoslav Republic of Macedonia, Turkey, Turkmenistan and Uzbekistan.

§ The year 2012 refers to year of publication. The year of data collection was not available for these countries.

### Baby-friendly hospitals

Table 4 further highlights that data on existing BFH were available for thirty-one out of fifty-three countries and twenty-eight of these reported having existing BFH (90 %). The proportion of BFH, however, varied considerably among WHO European countries, whereby less than 25 % of total hospitals with a maternity unit were ever designated as BFH in seventeen out of thirty-one countries (55 %) for which data were available on number of hospitals with maternity services. Only in eight countries had more than 50 % of hospitals ever been designated, with the highest percentage in Slovenia (93 %) and Norway (92 %) and the lowest reported in Italy (4 %), France (2 %) and Portugal (2 %). Three countries reported having no BFH, these being Greece, Iceland and Malta.

## Discussion

The current paper presents an important update of the current status of EBF practices, policy adoption and BFH in countries of the WHO European Region. Despite several health benefits and policy initiatives of optimal breastfeeding practices, the findings indicate that EBF remains far below the global recommendation of EBF and the national target in European countries. Moreover, the rates vary substantially across the region. Even though early initiation of breastfeeding rate is very high in some countries, EBF rates drop rapidly between 4 and 6 months, and are very low at 6 months of age. Overall, the present findings on breastfeeding practices confirm the result of an earlier analysis of breastfeeding practices^(^ ^33^ ^)^, i.e. lack of data harmonization, slow improvement in breastfeeding practices, low rates of EBF under 6 and at 6 months, and wide disparities in prevalence among the countries.

Although the benefits of EBF are widely regarded, global recommendations on optimal duration of EBF and introduction of complementary feeding are subject to debate in high-income countries^(^ ^42^ ^,^ ^43^ ^)^. Furthermore, there is a wide disparity in Europe concerning the adoption of the WHO recommendation of EBF for the first 6 months of life. According to a review done in 2009^(^ ^35^ ^)^, only eight European Union countries had adopted this recommendation, while others recommended the introduction of complementary feeding already between 4 and 6 months. It is important to notice that some experts and advisory committees are also not fully aligned with the WHO global recommendation of EBF. For instance, a medical position paper on complementary feeding by the European Society for Paediatric Gastroenterology Hepatology and Nutrition has concluded ‘exclusive breastfeeding for 6 months is a desirable goal and in any case complementary feeding should not be introduced before 17 weeks and not later than 27 weeks’^(^ ^42^ ^)^. Similarly, another scientific opinion on the appropriate age for introduction of complementary feeding of infants, by the European Food Safety Authority panel, has recommended to the European Commission that ‘the introduction of complementary food into the diet of healthy term infants in the EU between the age of 4 and 6 months is safe and does not pose a risk for adverse health effects’^(^ ^35^ ^)^. Similar recommendations have been practised in Belgium, Greece, Italy, Estonia and Poland^(^ ^42^ ^)^. These scientific opinions are not backed by systematic reviews, are not fully aligned with the current recommendation of EBF for the first 6 months, and may have a great deal of influence in EBF practices in many of the WHO European Region Member States.

Some key policy initiatives on breastfeeding and IYCF nutrition have been introduced in recent years. In 2006 the European Commission issued Directive 2006/125/EC^(^ ^44^ ^)^ on the composition and labelling of processed cereal-based foods for infants and young children, and Directive 2006/141/EC on the composition and labelling of infant formulae and follow-on formulae^(^ ^45^ ^)^. The very same year the European Charter on Counteracting Obesity was adopted in which the promotion of breastfeeding was strongly highlighted^(^ ^46^ ^)^. Furthermore the priority action areas of the Vienna Declaration on Nutrition and Noncommunicable Diseases in the Context of Health 2020^(^ ^47^ ^)^, the Global Strategy^(^ ^25^ ^)^, the WHO European Action Plan for Food and Nutrition Policy (2007–2012)^(^ ^48^ ^)^ and the new European Food and Nutrition Action Plan 2015–2020^(^ ^49^ ^)^ strongly emphasized the promotion and support for breastfeeding as a critical element for the development and appropriate nutritional status of children. Similarly, the Blueprint for Action for the Protection, Promotion and Support of Breastfeeding in Europe 2008 (revised) was launched to facilitate planning in policy and decision making^(^ ^26^ ^)^. These policy initiatives, along with the existence of a national policy, programmes and appropriate level of coordination, are some of the elected dimensions considered essential to increase optimal breastfeeding practices.

The findings of the present paper point to the fact that, where data are available, the majority of the WHO European Region Member States have policy tools in place. However, the actual results on breastfeeding practices do suggest that there might be some obstacles to the optimal implementation of policies and standards. An example is the UK, which has low EBF practices; only 1 % of infants are exclusively breastfed at 6 months. A recent paper described that supporting mothers in the UK who are exclusively breastfeeding at 1 week to continue breastfeeding until 4 months can be expected to save at least £11 million annually^(^ ^50^ ^)^. With the World Health Assembly targets, WHO Member States committed to increase the global rate of EBF in the first 6 months up to at least 50 % by 2025^(^ ^51^ ^)^. This is a target that many WHO European Member states are far from reaching.

A potential reason for this low rate in the UK is the non-existence of a national breastfeeding, IYCF or nutrition policy or committee, and furthermore no data to support if BFH have been implemented. There is increasing evidence suggesting that breastfeeding support and interventions are needed to enhance the rate of initiation, exclusivity and duration of breastfeeding^(^ ^52^ ^,^ ^53^ ^)^. The BFHI is one of these initiatives, and it has shown to be an effective strategy in increasing the initiation and, to some extent, duration of breastfeeding^(^ ^54^ ^,^ ^55^ ^)^. The proportion of hospitals in a country that are designated as BFH is one of the indicators for assessing country progress and priority on IYCF^(^ ^56^ ^)^. The data presented herein suggest that the proportion of BFH in each country is low, and in most instances the majority of the countries have no reported data.

### Limitations

The national data on breastfeeding practices were difficult to compare due to considerable cross-national variation; lack of standardized method and inconsistent use of definitions for data collection were also identified during the current analysis. Furthermore, data were not necessarily nationally representative and not always of sufficiently high quality. Although other studies have highlighted the need for data harmonization in European countries^(^ ^57^ ^,^ ^58^ ^)^, Europe still lacks a common strategy for the monitoring of breastfeeding practices. Therefore interpretations and comparisons should be done with great caution. With this being said, an initial analysis looking at any changes in breastfeeding prevalence over time was initiated. It was investigated if years of data collection differed in regard to breastfeeding practices above or below median rates. This was not the case, as the span of years was similar regardless of high or low breastfeeding rates.

## Conclusion

Breastfeeding practices within the WHO European Region, especially EBF, are currently not often in line with the WHO recommendations. There are marked differences between countries in breastfeeding practices, IYCF policy adoption and proportion of BFH in WHO European Member States. In addition, the findings of the present paper may help in raising awareness on the implementation and acceptance of WHO recommendations as well as attention to national breastfeeding monitoring systems.

## Appendix

***References for data on breastfeeding practice from WHO European Region Member States***

*Albania*

1. Institute of Statistics, Institute of Public Health & ICF Macro (2010) Albania Demographic and Health Survey 2008–2009. http://dhsprogram.com/pubs/pdf/FR230/FR230.pdf (accessed April 2014).

*Andorra*

No data

*Armenia*

1. National Statistical Service, Ministry of Health & ICF International (2012) Armenia Demographic and Health Survey 2010. http://dhsprogram.com/pubs/pdf/FR252/FR252.pdf (accessed April 2014).

2. UNICEF (2014) Global databases, based on Multiple Indicator Cluster Surveys (MICS), Demographic and Health Surveys (DHS) and other nationally representative surveys. http://data.unicef.org/nutrition/iycf (accessed April 2014).

*Austria*

1. Bundesministerium für Gesundheit, Familie und Jugend (Federal Ministry for Health, Family and Youth) (2007) Säuglingsernährung Heute 2006. Struktur- und Beratungsqualität an den Gerburtenkliniken in Österreich Ernährung von Säuglingen im ersten Lebensjahr (Infant Feeding Today 2006. Structure and quality advice to the maternity clinics in Austria for infants in the first year of life). http://www.bmg.gv.at/cms/home/attachments/2/8/5/CH1101/CMS1384785444563/langfassung_-_saeuglingsernaehrung_heute_-_endbericht_-_220707.pdf (accessed May 2015).

*Azerbaijan*

1. State Statistical Committee of Azerbaijan & Macro International Inc. (2008) Azerbaijan Demographic and Health Survey 2006. http://www.un-az.org/doc/AzDHS%20final.pdf (accessed April 2014).

2. World Health Organization (2009) Global Data Bank on Infant and Young Child feeding. Azerbaijan 2006. http://www.who.int/nutrition/databases/infantfeeding/countries/en/#top (accessed April 2014).

*Belarus*

1. National Statistical Committee of the Republic of Belarus & UNICEF (2013) Republic of Belarus Multiple Indicator Cluster Survey. Monitoring the Situation of Children and Women 2012. http://www.childinfo.org/files/MICS4_FinalReport_2012_Belarus_Eng.pdf (accessed April 2014).

*Belgium*

1. Kind and Gezin (Child and Family) (2012) Het kind in Vlaanderen (The child in Flanders). www.kindengezin.be/img/Het_kind_in_Vlaanderen_2012.pdf (accessed April 2014).

*Bosnia and Herzegovina*

1. UNICEF Office for Bosnia and Herzegovina (2013) Bosnia and Herzegovina Multiple Indicator Cluster Survey 2011–2012. http://www.unicef.org/bih/media_21363.html (accessed April 2014).

2. Directorate for Economic Planning, Ministry of Health and Social Welfare of Republic of Srpska & Ministry of Health of the Federation of Bosnia and Herzegovina (2007) Bosnia and Herzegovina Multiple Indicator Cluster Survey 2006. http://www.childinfo.org/files/MICS3_BiH_FinalReport_2006_Eng.pdf (accessed April 2014).

*Bulgaria*

1. Petrova S, Rangelova L, Duleva V *et al*. (2010) Current children’s breastfeeding practice in Bulgaria and determinants. *Bulg J Public Health* **4**, 11–26.

*Croatia*

1. Croatian National Institute of Public Health (2012) Croatian Health Service Yearbook 2011. http://hzjz.hr/wp-content/uploads/2013/11/Ljetopis_2011.pdf (accessed April 2014).

*Cyprus*

1. Organisation for Economic Co-operation and Development (2009) Family Database. Breastfeeding rates. http://www.oecd.org/els/family/43136973.xls (accessed April 2014).

*Czech Republic*

1. International Baby Food Action Network (2011) Report on the situation of infant and young child feeding in the Czech Republic. The Convention on the Rights of the Child, Session 57. May–June 2011. http://www.ibfan.org/art/IBFAN_CRC57-2011Czechrep.pdf (accessed April 2014).

*Denmark*

1. Statens Serum Institute (2013) Boernedatabasen (The Children’s Database). http://www.ssi.dk/Sundhedsdataogit/Registre/Bornedatabasen.aspx (accessed April 2014).

*Estonia*

No data

*Finland*

1. International Baby Food Action Network (2011) Report on the situation of infant and young child feeding in Finland. http://www.ibfan.org/art/IBFAN_CRC57-2011Finland.pdf (accessed April 2014).

*France*

No data

*Georgia*

1. The Georgia National Nutrition Survey, Steering Committee & UNICEF (2010) Report of the 2009 Georgia National Nutrition Survey. http://www.unicef.org/georgia/GNNS2009_eng_with_cover_edit.pdf (accessed April 2014).

*Germany*

1. Lange C, Schenk L & Bergmann R (2007) Verbreitung, Dauer und zeitlicher Trend des Stillens in Deutschland Ergebnisse des Kinder- und Jugendgesundheitssurveys (KiGGS) (Distribution, duration, and temporal trend of breastfeeding in Germany results of the Child and Adolescent Health Survey). *Bundesgesundheitsblatt Gesundheitsforschung Gesundheitsschutz* **50**, 624–633.

*Greece*

1. International Baby Food Action Network (2011) Report on the situation of infant and young child feeding in Greece. http://www.ibfan.org/art/IBFAN_CRC58_alternative_report-Greece.pdf (accessed April 2014).

*Hungary*

1. Organisation for Economic Co-operation and Development (2009) Family Database. Breastfeeding rates. http://www.oecd.org/els/family/43136973.xls (accessed April 2014).

*Iceland*

1. International Baby Food Action Network (2011) Report on the situation of infant and young child feeding in Iceland. http://www.ibfan.org/art/IBFAN_CRC58-2011_Iceland.pdf (accessed April 2014).

*Ireland*

1. University of Dublin, Trinity College Dublin, School of Nursing (2008) The National Infant Feeding Survey 2008. http://www.breastfeeding.ie/uploads/files/National_Infant_Feeding_Survey_2008.pdf (accessed April 2014).

*Israel*

1. Berger-Achituv S, Shohat T & Garty B (2005) Breastfeeding patterns in central Israel. *Isr Med Assoc J* **7**, 515–519.

*Italy*

1. Banderali G, Luotti D, Agostoni C *et al.* (2003) Monitoring breastfeeding rates in Italy: national surveys 1995 and 1999. *Acta Paediatr* **92**, 357–363.

*Kazakhstan*

1. Agency of Statistics, Kazakhstan & UNICEF (2012) Multiple Indicator Cluster Survey in the Republic of Kazakhstan 2010–2011. http://www.childinfo.org/files/Kazakhstan_MICS4_Final_Report_Eng.pdf (accessed April 2014).

*Kyrgyzstan*

1. National Statistical Committee of the Kyrgyz Republic, Ministry of Health & MEASURE DHS, ICF International (2013) Kyrgyz Republic Demographic and Health Survey 2012. http://dhsprogram.com/pubs/pdf/FR283/FR283.pdf (accessed April 2014).

2. UNICEF (2014) Global databases, based on Multiple Indicator, Cluster Surveys (MICS), Demographic and Health Surveys (DHS) and other nationally representative surveys. http://data.unicef.org/nutrition/iycf (accessed April 2014).

*Latvia*

1. Slimību profilakses un kontroles centrs (Centre for Disease Prevention and Control) (2011) Latvijas veselības aprūpes statistikas gadagrāmata (Latvian health statistics yearbook). http://www.spkc.gov.lv/veselibas-aprupes-statistika/ (accessed April 2014).

*Lithuania*

No data

*Luxembourg*

1. Le Gouvernement Du Grand-Duché De Luxembourg (Government of Luxembourg) (2010) L’alimentation de nos bébés. Enquête nationale sur l’alimentation des enfants de 4, 6 et 12 mois au Grand-Duché de Luxembourg en 2008 (Feeding our infants. National Nutrition Survey of children aged 4, 6 and 12 months in the Grand Duchy of Luxembourg). http://www.sante.public.lu/fr/catalogue-publications/rester-bonne-sante/alimentation/etude-alba-2008-alimentation-bebes/index.html (accessed April 2014).

*Malta*

1. Attard Montalto S, Borg H, Buttigieg-Said M *et al.* (2010) Incorrect advice: the most significant negative determinant on breast feeding in Malta. *Midwifery* **26**, e6–e13.

*Monaco*

No data

*Montenegro*

1. Statistical Office of Montenegro, Strategic Marketing Research Agency & UNICEF (2007) Montenegro Multiple Indicator Cluster Survey 2005. http://www.unicef.org/montenegro/MICS_3_PRINTED_TEXT.pdf (accessed April 2014).

*Netherlands*

1. Rijksinstituut voor Volksgezondheid en Milieu (National Institute for Public Health and Environment) (2013) Nationaal Kompas Volksgezondheid. Hoeveel moeders geven borstvoeding en neemt dit aantal toe of af? (National Public Health Compass. How many mothers do breastfeed and is this number increasing or decreasing?). http://www.nationaalkompas.nl/gezondheidsdeterminanten/leefstijl/borstvoeding/trend/ (accessed April 2014).

*Norway*

1. Lande B, Andersen LF, Baerug A *et al*. (2001) Infant feeding practices and associated factors in the first six months of life: the Norwegian Infant Nutrition Survey, 2003. *Acta Paediatr* **92**, 152–161.

*Poland*

1. Centrum Nauki o Laktacji (Centre for Lactation Science) (2013) Report on breastfeeding status in Poland 2013. http://www.kobiety.med.pl/cnol/index.php?option=com_content&view=article&id=153&Itemid=51&lang=en (accessed April 2014).

*Portugal*

1. Sandes AR, Nascimento C, Figueira J *et al.* (2007) Breastfeeding: prevalence and determinant factors. *Acta Med Port* **20**, 193–200.

*Republic of Moldova*

1. UNICEF (2014) Republic of Moldova. Monitoring the situation of children and women. Multiple Indicators Cluster Survey 2012. Summary Report. http://www.childinfo.org/files/Moldova_2012_MICS_Summary.pdf (accessed April 2014).

*Romania*

1. Ministry of Health (2005) Reproductive Health Survey Romania 2004. http://www.unece.org/unece/search?q=Reproductive+Health+Survey+Romania+2004 (accessed April 2014).

*Russian Federation*

No data

*San Marino*

No data

*Serbia*

1. Statistical Office of the Republic of Serbia & UNICEF (2011) Serbia Multiple Indicator Cluster Survey 2010. http://www.childinfo.org/files/MICS4_Serbia_FinalReport_Eng.pdf (accessed April 2014).

2. World Health Organization (2009) Global Data Bank on Infant and Young Child feeding. Serbia 2005–2006. http://www.who.int/nutrition/databases/infantfeeding/countries/en/#top (accessed April 2014).

*Slovakia*

1. Bratislava National Health Information Center (2011) Ambulantá Starostlivost´o deti a dorast v SR 2010 (Outpatient care for children and adolescents in Slovakia). http://www.nczisk.sk/Documents/publikacie/2010/zs1126.pdf (accessed April 2014).

*Slovenia*

No data

*Spain*

1. Instituto Nacional de Estadistica (National Institute of Statistics) (2013) Tipo de lactancia según clase social basada en la ocupación de la persona de referencia. 2011–2012 (Type of feeding according to social class based on the occupation of the reference person. 2011–2012). http://www.ine.es/ss/Satellite?c=INESeccion_C&p=1254735110672&pagename=ProductosYServicios%2FPYSLayout&cid=1259926457058&L=1 (accessed April 2014).

*Sweden*

1. Socialstyrelsen (Ministry of Social Welfare) (2013) Amning och föräldrars rökvanor – Barn födda 2011 (Breastfeeding and parents smoking habits – Children born in 2011). http://www.socialstyrelsen.se/publikationer2013/2013-9-18 (accessed April 2014).

*Switzerland*

1. World Health Organization (2009) Global Data Bank on Infant and Young Child feeding. Switzerland 2003. http://www.who.int/nutrition/databases/infantfeeding/countries/en/#top (accessed April 2014).

*Tajikistan*

1. Statistical Agency, Ministry of Health & MEASURE DHS, ICF International (2012) Tajikistan Demographic and Health Survey 2012. Preliminary Report. http://dhsprogram.com/pubs/pdf/PR26/PR26.pdf (accessed April 2014).

*The former Yugoslav Republic of Macedonia*

1. UNICEF (2011) Republic of Macedonia. Multiple Indicator Cluster Survey 2011. http://www.childinfo.org/files/Macedonia_FYRM_2011_MICS_(National_and_Roma_Settlements)_revised_2014.pdf (accessed April 2014).

*Turkey*

1. The Scientific and Technological Research Council of Turkey (2009) Turkey Demographic and Health Survey 2008. Preliminary Report. http://www.hips.hacettepe.edu.tr/eng/tdhs08/TDHS-2008_Preliminary_Report.pdf (accessed April 2014).

*Turkmenistan*

1. Childinfo (2007) Infant and young child feeding. http://www.childinfo.org/breastfeeding_countrydata.php (accessed April 2014).

*Ukraine*

1. State Statistics Service, Ukrainian Institute for Social Reforms, Statinformconsulting, *et al*. (2013) Ukraine Multiple Indicators Cluster Survey 2012. Final Report. http://www.unicef.org/ukraine/Ukraine_MICS_FinalReport_ENG.pdf (accessed April 2014).

*UK*

1. Health and Social Care Information Centre (2012) Infant Feeding Survey – UK 2010 (NS). http://www.hscic.gov.uk/article/2021/Website-Search?productid=9569&q=infant+feeding+survey&sort=Relevance&size=10&page=1&area=both (accessed April 2014).

*Uzbekistan*

1. Cabinet of Ministers, State Statistical Committee, UNICEF *et al*. (2007) Uzbekistan Multiple Indicator Cluster Survey 2006 Final Report. http://www.childinfo.org/files/MICS3_Uzbekistan_FinalReport_2006_Eng.pdf (accessed April 2014).
